# Case Report: Chicken bone-induced perforation of duodenal diverticulum in the third portion successfully treated by endoscopy

**DOI:** 10.3389/fmed.2025.1642959

**Published:** 2025-10-08

**Authors:** Hui-hui Zhou, Cui-mei Ma, Yan Wang, Yao-wen Zhang

**Affiliations:** ^1^Department of Gastroenterology, Affiliated Hospital of Jining Medical University, Jining, China; ^2^Endoscopy Department, Affiliated Hospital of Jining Medical University, Jining, China

**Keywords:** duodenal diverticulum, foreign body, perforation, the third portion of the duodenum, endoscopic treatment

## Abstract

Duodenal diverticulum (DD) perforation is a rare but severe clinical emergency with a reported mortality rate as high as 30%. DD most commonly occurs in the second portion of the duodenum (D2), particularly in the periampullary region, accounting for 78.3% of cases. In contrast, diverticula in the third portion of the duodenum (D3) are relatively rare. Traditional treatment methods include conservative therapy, percutaneous drainage, and surgical intervention. However, with advancements in gastrointestinal endoscopy, endoscopic treatment has emerged as a viable option. A 71-year-old male patient presented with upper abdominal pain. Laboratory tests revealed elevated white blood cell count and C-reactive protein levels. Abdominal CT showed a perforated diverticulum in D3 with a suspected foreign body. After 5 days of conservative treatment with no improvement, endoscopic intervention was performed. Using a gastroscope equipped with a transparent cap, foreign body forceps, the embedded chicken bone and surrounding debris were removed, and the fistula was closed with metallic clips. Follow-up CT scans showed significant improvement, with no recurrence of symptoms at 18 months. The successful endoscopic treatment of this D3 diverticulum perforation highlights the potential of endoscopic therapy in managing complex duodenal diseases. Compared to traditional surgery, endoscopic treatment offers minimal invasiveness, faster recovery, and fewer complications. This case expands the application of endoscopic techniques to D3 perforations, providing valuable experience for future similar cases.

## 1 Introduction

Duodenal diverticulum(DD) perforation is a rare but severe clinical emergency, with a reported mortality rate as high as 30% (1, 2). On this basis, duodenal perforation caused by ingested foreign bodies is even rarer. DD most commonly occur in the second portion of the duodenum (D2), especially in the periampullary, accounting for 78.3% of cases (3). In contrast, diverticula in the third portion of the duodenum (D3) are relatively rare. In terms of treatment, conservative management is often employed for stable patients without signs of peritonitis. However, for patients who fail conservative therapy, percutaneous drainage or surgical intervention is required (4). With the continuous advancement of gastrointestinal endoscopy techniques, there have been successful case reports of endoscopic treatment for D2 diverticulum perforation (4, 5). Building on this foundation, we present the first case report of a patient with D3 diverticulum perforation caused by a foreign body that was successfully treated by endoscopy.

## 2 Case description

**Figure 1:**
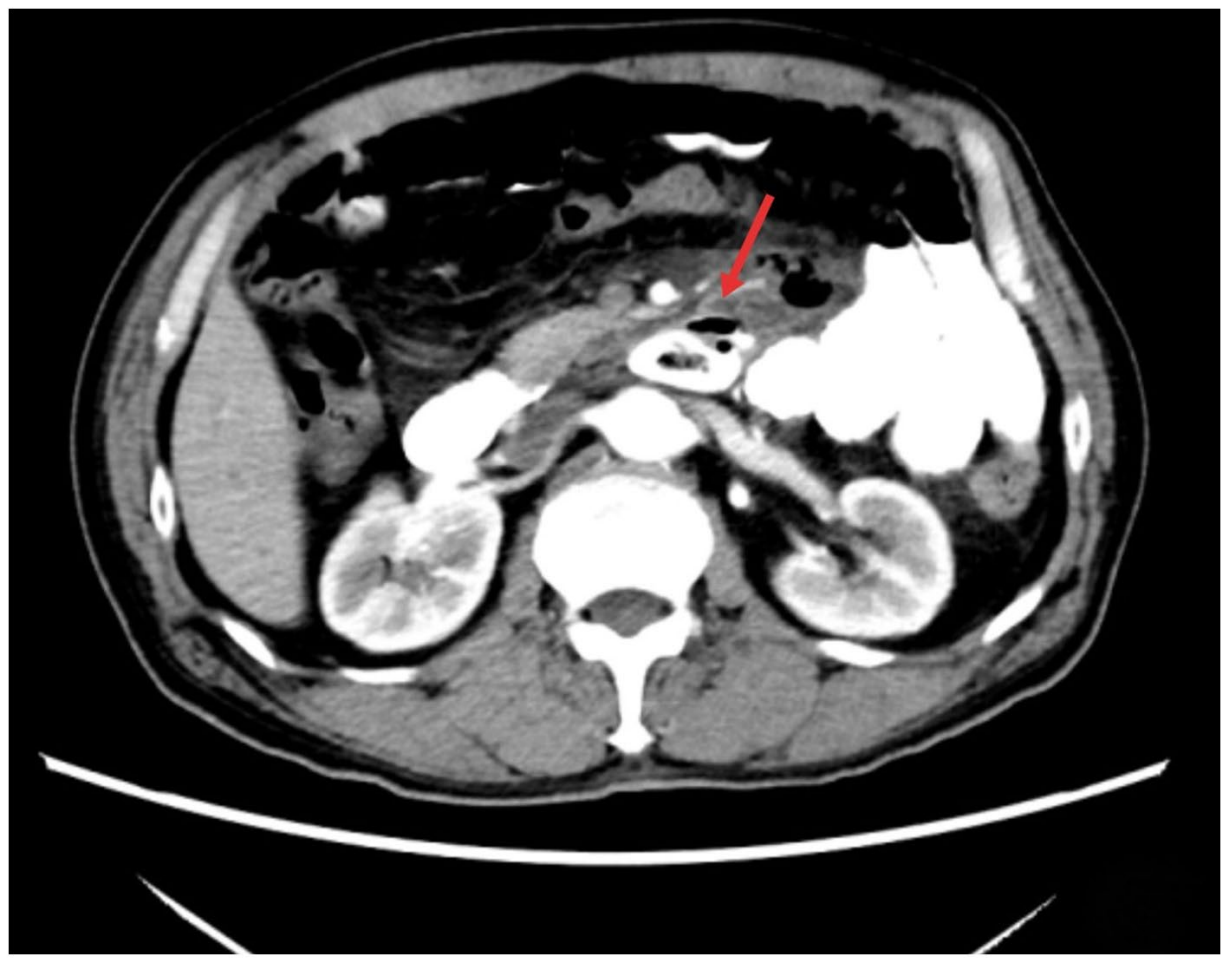
Abdominal CT shows a cystic shadow is seen in the third portion of the duodenum, with a hollow high-density area inside. A localized gas shadow is observed in front (indicated by the arrow).

**Figure 2:**
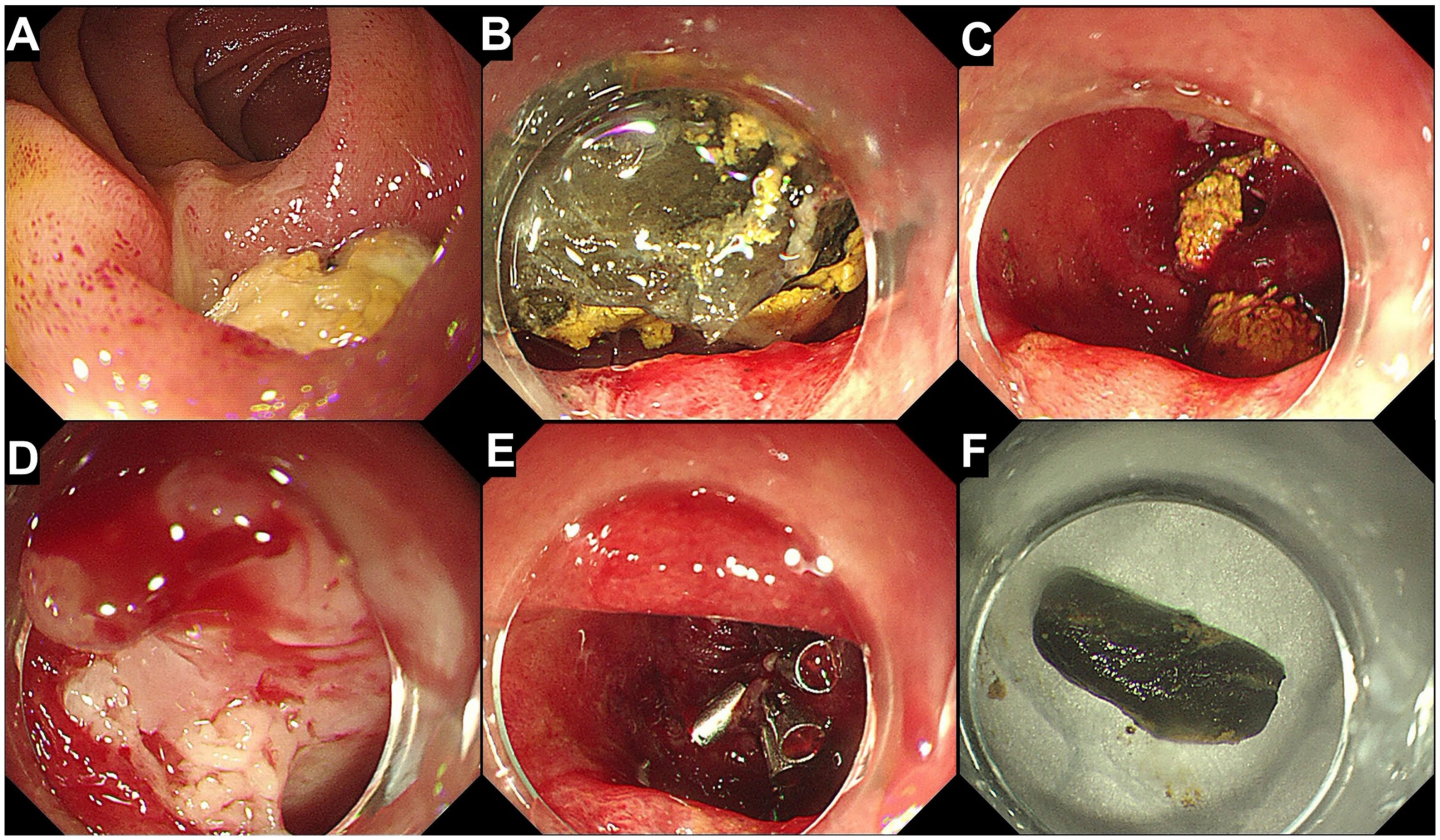
Endoscopic removal of the foreign body and closure of the fistula. **(A)** Endoscopic view of a diverticulum in the third portion of the duodenum, containing a food mass. **(B)** Anterior view of the diverticulum showing food debris and a hard foreign body filling the diverticulum. **(C)** After removal of the food debris and the foreign body, the wide base of the diverticulum is visible. **(D)** A fistula is observed at the base of the diverticulum, with surrounding mucosa showing signs of congestion and edema. **(E)** The fistula is closed using five metallic clips. **(F)** The extracted chicken bone.

**Figure 3:**
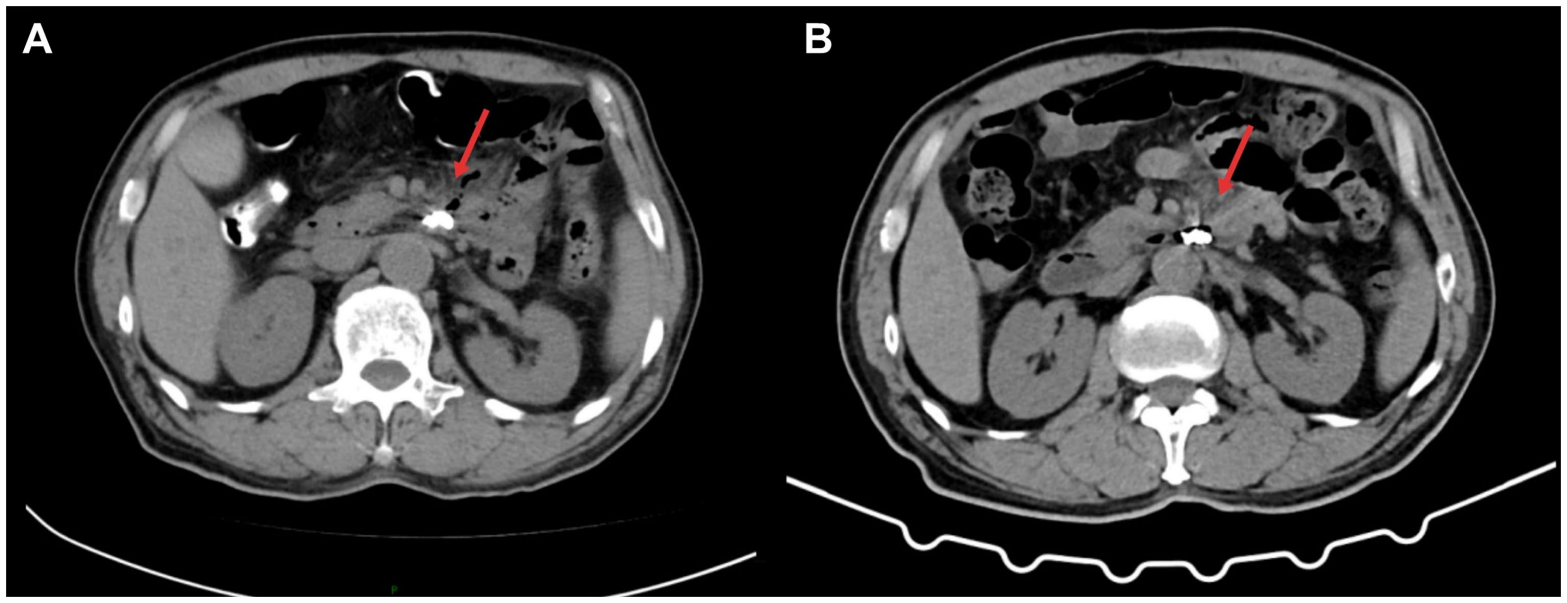
Postoperative follow-up. **(A)** Abdominal CT: Post-treatment appearance of a duodenal diverticulum in the third portion with perforation. Metallic clips are visible in the surgical area, and there is a reduction and absorption of the gas shadow anterior to the cystic structure compared to before (indicated by the arrow). **(B)** Abdominal CT: Post-treatment appearance of a third portion duodenal diverticulum with perforation. Metallic clips are visible in the surgical area, and there is no significant display of the gas shadow anterior to the cystic structure (indicated by the arrow).

A 71-year-old male patient presented to our hospital with a chief complaint of “upper abdominal pain for 3 days.” The pain was intermittent, predominantly located in the left upper abdomen, and was severe in intensity, radiating to the back. It was accompanied by abdominal distension and reduced bowel movements and flatus. The patient has a 30-year history of being a hepatitis B virus (HBV) carrier, a 10-year history of surgery for a right inguinal hernia, and a 2-year history of surgery for varicose veins in the right lower limb. On admission physical examination: T 36.5 °C, tenderness in the upper abdomen, localized muscle tension in the left upper abdomen, and positive rebound tenderness. Laboratory tests revealed: WBC 18.75 × 10^9^/L, NEUT% 87; CRP167.2 mg/L, D-dimer 2.21 mg/L. Liver and kidney function, urinalysis, and myocardial injury markers were within normal limits. Abdominal contrast-enhanced CT revealed a diverticulum with perforation of D3; high-density shadow within the diverticulum: foreign body to be excluded (Figure 1). The patient was managed conservatively with fasting, gastrointestinal decompression, acid suppression, antibiotic therapy, and parenteral nutrition support for 5 days; however, his symptoms did not improve. After obtaining informed consent from the patient, endoscopic removal of the foreign body from D3 diverticulum and closure of the fistula were performed under general anesthesia with endotracheal intubation and monitoring. The surgical instruments included a standard gastroscope (OLYMPUS GIF-Q260J), a foreign body forceps (OLYMPUS FG-44NR-1), and a transparent cap (OLYMPUS D-201-11304). The gastroscope, equipped with a transparent cap, was advanced to the third portion of the duodenum, where a diverticulum collar approximately 1.6 cm × 1.0 cm in size was observed, filled with a large amount of food debris and chicken bones, with a broad base. We used the foreign body forceps to remove the chicken bones embedded in the tissue and the surrounding food debris in multiple sessions. Two ulcers were seen at the base of the diverticulum, with the larger one showing a fistula at the base, surrounded by congested and edematous mucosa that was fragile. Five metallic clips (Micro-Tech(Nanjing), China, ROCC-D-26-195) were used to close the fistula (Figure 2). On postoperative day 4, a follow-up abdominal CT scan showed the appearance after treatment of D3 diverticulum and perforation, with visible metallic clips in the surgical area and a reduction in gas compared to before(Figure 3A). The complete blood count showed that WBC and CRP had returned to normal levels, and gastrointestinal decompression was discontinued. Antibiotics were stopped on postoperative day 6, clear-fluid feeding was initiated the same evening and was fully tolerated without pain or rise in temperature. The patient was discharged on day 8 on a step-wise dietary progression (clear → full-liquid → low-residue soft → regular), remained afebrile (≤37.2 °C), and reported no abdominal pain throughout. On postoperative day 16, a follow-up abdominal CT scan showed the appearance after treatment of D3 diverticulum and perforation, with visible metallic clips and no visible gas (Figure 3B). The patient has been followed up for 18 months without recurrence of abdominal pain or other significant discomfort.

Ethical approval for this case was obtained from the Medical Ethics Committee of the Affiliated Hospital of Jining Medical University. Written informed consent for the publication of anonymized information was obtained from the patient and their authorized representative.

## 3 Discussion

DD was first described by Chromel in 1710 (6), and endoscopic diagnosis was not reported until Ryan et al. in 1984 (7). DD are most commonly located in D2, particularly in the periampullary region, accounting for 78.3% of cases (3). Diverticula in D3 are relatively rare. The vast majority of DD are asymptomatic or incidentally detected during imaging studies and do not require specific treatment. Although complications of DD, including upper gastrointestinal bleeding, perforation, and diverticulitis, are uncommon, they can be severe when they occur and often necessitate intervention. Perforation is one of the rarest and most severe complications (1). The etiology of duodenal diverticulum perforation is multifactorial, with the main causes including diverticulitis, bezoars, iatrogenic injury, ulceration, trauma, foreign bodies, and unknown causes (5, 8). In the present case, the perforation occurred in D3 due to a chicken bone embedded in the tissue, a scenario not previously reported. In resource-limited settings, prompt access to endoscopy or CT imaging may not be available, resulting in delayed patient presentation and diagnosis and allowing a potentially salvageable foreign-body perforation to become a fatal event (9). Therefore, clinicians must maintain a high index of suspicion and advocate for timely evaluation and treatment whenever a sharp or large foreign body is suspected, even if initial symptoms appear to resolve.

The traditional treatment methods for DD perforation mainly include conservative therapy, percutaneous drainage, and surgical treatment. For uncomplicated duodenal diverticulitis and perforation, conservative treatment may be successful, thus avoiding surgical overtreatment and its potential serious complications, except in cases of peritonitis or sepsis (10). Conservative treatment usually consists of bowel rest, intravenous fluid resuscitation, and the use of broad-spectrum antibiotics (11). However, for patients who fail conservative treatment, percutaneous drainage or surgical treatment can be considered. As reported in the literature, approximately 34% of patients are managed non-operatively, with a preference for conservative treatment; however, 31% of these patients initially treated conservatively require subsequent surgical intervention (4). Surgical approaches vary widely, ranging from simple diverticulectomy to Roux-en-Y gastric diversion or even Whipple’s procedure, depending on tissue friability and the size of the diverticular collar. Especially for diverticula located in D3/D4, partial duodenectomy with end-to-end or end-to-side duodenojejunostomy may be more appropriate due to anatomical considerations (4). However, surgical treatment is highly invasive, associated with a long postoperative recovery period, and may not be suitable for elderly patients or those with comorbidities that preclude surgery. With the continuous advancement of gastrointestinal endoscopy techniques, the success rate of endoscopic repair for duodenal perforations has reached as high as 90.4%. Under the operation of experienced endoscopists, many duodenal perforations can be managed with endoscopic repair, which has become a viable first-line treatment option, especially for patients for whom surgical treatment is not feasible (12). In the present case, the patient’s CRP level was as high as 167 mg/L, which is significantly higher than the commonly reported range of less than 100 mg/L for patients with DD perforation in previous literature (4). This markedly elevated CRP value suggests a more severe inflammatory response in the patient. Despite undergoing 5 days of conservative treatment, the patient’s symptoms did not show significant improvement, which may be related to the residual foreign body within the diverticulum and the persistent irritation and infection caused by the embedded foreign body. Therefore, considering the above circumstances, the patient requires further intervention to effectively control the inflammation and address the perforation. After obtaining informed consent from the patient, we initially opted for endoscopic treatment. Although the diverticulum was located in D3, which is difficult to reach with a conventional endoscope, according to the recommendations of the “Consensus on the endoscopic management of foreign bodies in the upper gastrointestinal tract in China” (13), enteroscopy could also be considered. However, we still attempted to use a gastroscope for the procedure and successfully reached the lesion by repeated insufflation and abdominal compression. Given the small collar and large base of the diverticulum, we used a transparent cap to assist in advancing the scope to maintain a good visual field, successfully removed the foreign body, and closed the fistula with metallic clips. The patient had an uneventful recovery. For such patients, endoscopic treatment enables accurate localization of the diverticulum and foreign body, assessment of diverticulum morphology and perforation size, and subsequent removal of the foreign body with foreign body forceps and endoscopic repair of the perforation, highlighting the advantages of endoscopic therapy. Previous reports have mainly focused on endoscopic treatment of perforated D2 diverticula (5). Apart from the case of D3 diverticulum bleeding reported by Chen et al. (14), this is the first reported case of successful endoscopic removal of a foreign body embedded in D3 diverticulum and treatment of the perforation.

The successful treatment of this case not only highlights the advantages of endoscopic therapy in managing D3 diverticulum perforation but also further expands the application scope of endoscopic techniques in complex duodenal diseases. Compared with traditional surgery, endoscopic therapy has the advantages of minimal invasiveness, faster recovery, and fewer complications, providing valuable experience and reference for the treatment of similar cases in the future.

However, important limitations must be acknowledged. The favorable outcome was contingent on a single, highly experienced therapeutic endoscopy team and readily available fluoroscopy and intensive-care support—resources that may be unavailable in low-resource settings. Additionally, we did not perform repeat endoscopy to document mucosal healing, relying instead on imaging and clinical follow-up. Prospective series with longer follow-up and objective healing criteria are required before these findings can be generalized.

### 3.1 Patient perspective

During the 18-month follow-up, the patient expressed great relief that his abdominal pain had completely resolved and he could resume normal diet and daily activities. He reported a strong preference for the minimally invasive endoscopic approach over open surgery after being informed of the potential morbidity associated with laparotomy in a 71-year-old with multiple comorbidities. He also emphasized that, in his rural community, rapid access to endoscopy and CT is limited; therefore, he now actively shares his experience to encourage relatives and neighbors to seek early hospital evaluation when foreign-body ingestion is suspected, even if symptoms appear mild or transient.

## Data Availability

The original contributions presented in the study are included in the article/supplementary material, further inquiries can be directed to the corresponding author.

## References

[1] Banal C Stevens C. Perforated duodenal diverticulum treated conservatively. BMJ Case Rep. (2024) 17:e259643. doi: 10.1136/bcr-2024-259643, PMID: 39214589

[2] Teven CM Grossman E Roggin KK Matthews JB. Surgical management of pancreaticobiliary disease associated with juxtapapillary duodenal diverticula: case series and review of the literature. J Gastrointest Surg. (2012) 16:1436–41. doi: 10.1007/s11605-012-1856-z, PMID: 22392090

[3] Yilmaz E Kostek O Hereklioglu S Goktas M Tuncbilek N. Assessment of duodenal diverticula: computed tomography findings. Current Med Imaging Rev. (2019) 15:948–55. doi: 10.2174/1573405614666180904123526, PMID: 32008522

[4] Kapp JR Müller PC Gertsch P Gubler C Clavien PA Lehmann K. A systematic review of the perforated duodenal diverticula: lessons learned from the last decade. Langenbeck's Arch Surg. (2022) 407:25–35. doi: 10.1007/s00423-021-02238-1, PMID: 34164722 PMC8847262

[5] Tadokoro T Oishi K Namba Y Bekki T Okimoto S Mukai S. Duodenal diverticula perforation caused by an impacted bezoar successfully treated by endoscopic drainage and lithotripsy: a case report and literature review. Clin Case Rep. (2022) 10:e6619. doi: 10.1002/ccr3.6619, PMID: 36419578 PMC9676119

[6] Chitambar IA Springs C. Duodenal diverticula. Surgery. (1953) 33:768–91. PMID: 13056877

[7] Ryan ME Hamilton JW Morrissey JF. Gastrointestinal hemorrhage from a duodenal diverticulum. Gastrointest Endosc. (1984) 30:84–7. doi: 10.1016/s0016-5107(84)72325-x, PMID: 6425109

[8] Duarte B Nagy KK Cintron J. Perforated duodenal diverticulum. Br J Surg. (1992) 79:877–81. doi: 10.1002/bjs.1800790907, PMID: 1422745

[9] Pranavan S Mayorathan U Munasinghe BM. A fatal aorto-oesophageal fistula due to a mutton bone: A case report. Int J Surg Case Rep. (2023) 108:108478. doi: 10.1016/j.ijscr.2023.108478, PMID: 37421771 PMC10382798

[10] Álvarez-García M Milán Pilo MV Otegui L Calvo Hernández R Polo B Bosch Esteva O. To considerate perforation of duodenal diverticula. Rev Esp Enferm Dig. (2025). 9. doi: 10.17235/reed.2024.10955/2024 39784684

[11] Moysidis M Paramythiotis D Karakatsanis A Amanatidou E Psoma E Mavropoulou X. The challenging diagnosis and treatment of duodenal diverticulum perforation: a report of two cases. BMC Gastroenterol. (2020) 20:5. doi: 10.1186/s12876-019-1154-2, PMID: 31914931 PMC6951008

[12] Williams J Joshi H Schwartz M Kalola A Mercado A Saracco B. Endoscopic repair of duodenal perforations, a scoping review. Surg Endosc. (2024) 38:4839–45. doi: 10.1007/s00464-024-11133-x, PMID: 39143329 PMC11362252

[13] Chinese Society of Digestive Endoscopy Consensus on the endoscopic management of foreign bodies in the upper gastrointestinal tract in China (2015, Shanghai). Chin J Digest Endosc. (2016) 33:19–28. doi: 10.3760/cma.j.issn.1007-5232.2016.01.003

[14] Chen YY Yen HH Soon MS. Impact of endoscopy in the management of duodenal diverticular bleeding: experience of a single medical center and a review of recent literature. Gastrointest Endosc. (2007) 66:831–5. doi: 10.1016/j.gie.2007.06.001, PMID: 17905030

